# How can we support research participants who stop taking part? Communications guidance developed through public-researcher collaboration

**DOI:** 10.1186/s40900-024-00572-4

**Published:** 2024-04-18

**Authors:** William J. Cragg, Liam Bishop, Rachael Gilberts, Michael Gregg, Terry Lowdon, Mary Mancini, Clara Martins de Barros, Pete Wheatstone

**Affiliations:** 1 Clinical Trials Research Unit, Leeds Institute of Clinical Trials Research, University of Leeds, Leeds, UK; 2 Public contributor, Leeds, UK

**Keywords:** Participant communication, Withdrawal, Participation changes

## Abstract

**Background:**

Research study participants can stop taking part early, in various circumstances. Sometimes this experience can be stressful. Providing participants with the information they want or need when they stop could improve participants’ experiences, and may benefit individual studies’ objectives and research in general. A group of public contributors and researchers at the Clinical Trials Research Unit, University of Leeds, aimed to develop a communication template and researcher guidance. This would address how to provide information sensitively around the time when participants stop or significantly reduce their level of participation.

**Methods:**

The project lead used scoping review methods to identify relevant prior evidence and derive a list of potential information topics to communicate to participants who stop taking part. The topic list was reviewed by research professionals and public contributors before finalisation. Further public contributors were identified from a range of networks. The contributors formed a ‘development group’, to work on the detail of the planned resources, and a larger ‘review group’ to review the draft output before finalisation. The involvement was planned so that the development group could shape the direction and pace of the work.

**Results:**

The literature review identified 413 relevant reports, resulting in 94 information topics. The review suggested that this issue has not been well explored previously. Some evidence suggested early-stopping participants are sometimes excluded from important communications (such as study results) without clear justification. The development group agreed early to focus on guidance with reusable examples rather than a template. We took time to explore different perspectives and made decisions by informal consensus. Review group feedback was broadly positive but highlighted the need to improve resource navigability, leading to its final online form.

**Conclusions:**

We co-developed a resource to provide support to research participants who stop taking part. A strength of this work is that several of the public contributors have direct lived experience of stopping research participation. We encourage others to review the resource and consider how they support these participants in their studies. Our work highlights the value of researchers and participants working together, including on complex and ethically challenging topics.

**Supplementary Information:**

The online version contains supplementary material available at 10.1186/s40900-024-00572-4.

## Abstract (plain language summary)

Participants in research sometimes stop taking part early. This can sometimes be stressful or difficult for them. Giving them information they want or need around that time could help them and the research. Public contributors and researchers worked together on this project. We wanted to help researchers get information to research participants who stop taking part. Some of the public contributors had experiences of stopping research participation early.

The project lead first made a rough plan for the project, with public contributors’ help. He left the plan open so the public contributors could help shape the project. The project lead searched for relevant information in published literature. This search showed there has not been much work before on how to help participants who stop taking part. He used the search results to make a list of topics that could be useful to give participants who stop taking part. He asked public contributors and researchers to review the list.

Public contributors then joined one of two groups. A smaller group worked on the detail of the planned guidance. A larger group reviewed the draft guidance.

The smaller group worked together to make the final guidance in six online meetings. The guidance includes example wording for others to use in their own participant communications. The reviewer group generally liked the guidance but had comments on making it easier to use. The final resource is available online and a link is in the references to this article.

**Supplementary Information:**

The online version contains supplementary material available at 10.1186/s40900-024-00572-4.

## Background

It is universally accepted that participants in clinical trials and other research studies can withdraw their informed consent at any time after having given it. They can do this without having to give a reason, and without their usual care being affected.

There is evidence that ending participation in a study can be difficult for some participants. Some feel unsupported or even ‘abandoned’ [1, 2]. Some of us as authors of this paper have our own experiences of ending participation, which have sometimes been negative. For example, some of us have found that it has not always been easy to communicate that we wanted to stop taking part. Once we had stopped taking part, we did not always have good communication from research staff about what was happening next, or about things like what might happen to incentives previously offered in return for our participation.

Participants might feel more supported if they have access to information they want or need around the time they stop taking part. This may improve their overall experiences taking part and increase the chance of them taking part in research again in future. It also aligns with evidence on participants’ research experiences, and the suggestion that participants should get the information they need at different times, as they progress through a study [3].

Providing better quality information might also help research studies’ objectives. The Persevere project (PRincipleS for handling end-of-participation EVEnts in clinical trials REsearch [4]) was set up through the UK Clinical Research Collaboration Registered Clinical Trials Unit Network. Its aim was to establish standard approaches (via a set of guiding principles) to prepare for and manage the potential complexity in how participants might stop, reduce or change their participation in research studies. Part of the motivation of that project was to ensure participants’ experiences of changing their level of participation are as good as they can be. The Persevere project’s methods and outputs will be reported separately.

One of the Persevere principles, coded ‘O2’, states that it should mainly be participants who decide on the nature and extent of changes in their participation [5]. In many studies, participants might have options about which study activities they continue and which they stop. The main exception to this principle is where someone else advises that some aspect of study participation should stop – for example, where a participant’s doctor decides that study treatment should stop. The Persevere guidance states that “[t]hese decisions by others should only apply to relevant aspects of participation, and not beyond these” [5].

There is evidence to suggest that participants most often stop taking part for practical reasons or because they can no longer accommodate study participation in their lives, rather than because they no longer support the research aims [6–8]. They might therefore be interested in continuing participating with less commitment (for example continuing to allow data to be collected from routine healthcare visits), if they were given the chance. There might be advantages for them – in a broad sense – from such further, reduced participation.

Widespread anecdotal evidence observed through the Persevere project suggests that participants are not always given that chance, and that participants’ expression of a general wish to stop some aspects of participation is sometimes interpreted as a wish to stop all participation.

Participants’ decisions about changing their level of participation should be informed and freely-made, in the same way as their initial consent to take part. It seems likely that pre-study information about stopping participation is not as good as it could be [9, 10], possibly reducing the ‘informedness’ of participants’ decisions. Improving pre-participation information could therefore be important. There may be a role for reminding participants about key information while they are making their final decision about how their want their participation level to change [11].

Confirmatory information given *after* a participant changes their level of involvement can include details of exactly how participants’ involvement in the study has changed. This can give participants clarity about their choices and about how research staff have understood participants’ wishes. Although it is after the participation change, there is some chance that this clarity could help make sure participants are involved in decisions about the nature and extent of their participation change. The idea of providing information is supported by another Persevere principle, coded ‘O7’ [12]. This states that stopping or reducing participation does not mean participants cannot get information they might want or need.

There is guidance for researchers on communicating with participants at the end of a study [13], but we are not aware of any guidance aimed to help participants who stop taking part early. It may be that participants who stop taking part have specific information needs – distinct from the needs of those who ‘complete’ their participation in a study – that have not been well considered so far.

Because of the rules around withdrawal of consent, and the training researchers get about this, researchers might feel it is no longer appropriate to engage with participants once they have stopped taking part. This might mean participants do not get the information they want or need.

During the Persevere project, participant information at the time of stopping was identified as an area where more guidance would be useful. Researchers from the Persevere project group therefore set out to develop some, in collaboration with public contributors.

We were clear from the outset that this information for participants would be given after a decision to stop or significantly reduce participation, and would be intended to provide information, not to change minds. The communication would only be aiming to provide information relevant to the participant’s change in involvement level, around the time of the change. Information would only be provided after this time if participants opted into it. Such further communications were outside of our scope.

Involving patients and the public in this work was essential. The Persevere researchers wanted to co-design an output, to bring together researchers’ and potential/actual participants’ perspectives, including those who have experience of stopping their participation in research. The output would not have been valid otherwise.

## Methods

### Overview

We co-developed a resource for researchers about creating written communications for research participants who stop taking part. The initial aim was to develop a template communication with guidance about how to use it. We drew on our experiences as research participants and as people involved in creating or reviewing information for participants. We used the outputs from a scoping literature review to guide our discussions.

### Literature review

A literature review was carried out by the project lead (WJC) to find reports addressing the following broad questions in the context of adult research participants who can consent for themselves:

- What information should research participants get around the time they finish taking part in a research study?
- Of the information topics/items identified for communication at the end of a study, which are specific to participants who end participation early?
- How does each information topic/item need to be changed or made more specific for participants who end participation early?
- What is the best method of getting this information to participants who stop taking part?

WJC followed the stages of scoping review methodology [14]. Elements required by the PRISMA-ScR checklist [15] have informed this current report. WJC developed a search strategy by devising search terms across a range of relevant concepts, then combining these groups of terms in different ways. Searches were carried out in PubMed, Ovid Medline and Embase, and CINAHL. The PubMed search strategy is available with this article as Supplementary material.

No limitations were built into the search strategy regarding date of publication, study type, study setting or publication language. Search results were excluded if they did not contain information relevant to the search objectives, or they did not relate to the relevant setting (e.g. reports about studies with child participants).

WJC carried out Google searches based on the same terminology lists to find materials not published in peer-reviewed journals. He added to the final results reports that were already known to him, or that were identified ad hoc during the literature review period, or that were found through reference and citation searching on any directly relevant reports.

WJC collected data from all relevant reports using a pre-specified data collection form. This included recording which topics each report suggested should be provided to research participants at the end of their involvement in a study.

An inclusive approach was taken to identifying information topics, meaning that all potential ideas were included, even if the report authors had not suggested them to be specifically relevant for participants who stop taking part in studies. Identified reports were categorised as ‘key paper’ (directly relevant to the search objectives) and ‘indirectly relevant’ (containing relevant ideas, but not directly about a topic relevant to the search objectives).

Due to the exploratory nature of the search, WJC did not contact authors for further information, or formally assess the quality of identified reports.

### Developing a list of information topics for participants who stop taking part

WJC developed a ‘topic list’, intended to contain all possible topics or information items that might be relevant to communicate when a research participant stops taking part. The list was produced using the results of the literature search and two other sources: 1) the Health Research Authority’s guidance on end of study information [13], and 2) topics arising from the Persevere project guidance [4].

The topic list was shared for feedback with interested research professionals and public contributors involved in the Persevere project, as well as research professionals linked to the Trial Methodology Research Partnership retention and communications subgroups [16].

### Identifying public contributors for the group work

Public contributors were sought to form two separate groups: a ‘development group’ to work in detail on a template communication with accompanying guidance, and a ‘review group’ to conduct a separate review of the draft template and guidance. This way of working borrowed from the ‘user-testing’ model of developing patient information suggested by others [17].

A Patient and Public Involvement plan was developed, aiming to find a diverse group of contributors who, if possible, had experience of stopping research participation early. Public contributors linked to the Clinical Trials Research Unit (CTRU) at the University of Leeds helped develop the plan and agree the final membership of the two groups. The opportunity to be involved was advertised widely, given that the activity was not specific to one health condition. We asked for expressions of interest to be submitted via a form in Jisc Online Surveys [18]. Potential contributors could express an interest in joining the development group, or the review group, or either. Selection was based on finding individuals with relevant experiences (particularly experiences of stopping research participation) and on establishing a group of public contributors with diverse characteristics overall. All interested individuals were given the chance to stay in touch with the outcomes of the project, regardless of whether they were chosen to be involved.

The project researchers were also interested to get input from research nurses to inform the project, as they represent a group that would often be involved in delivering the information to research participants. An invitation to contribute to the project was disseminated to several research nurses already known to the project lead, and via Twitter (now X) with encouragement for the message to be shared.

### Developing the resource

We planned to develop the resource through meetings of the ‘development group’. Meetings would not be recorded, but the project lead would take notes to inform resource development. The meetings would follow a roughly pre-defined plan, but with flexibility to deal with any unexpected challenges, and so that the group members could help shape the process. The topic list would serve as a starting point for the discussions. Only the first two meetings were planned in detail in advance, with the rest left open to discussion and agreement with group members.

Once the initial draft of the resource was ready, we would invite the ‘review group’ to give feedback.

## Results

### Literature review and topic list development

**Fig. 1:**
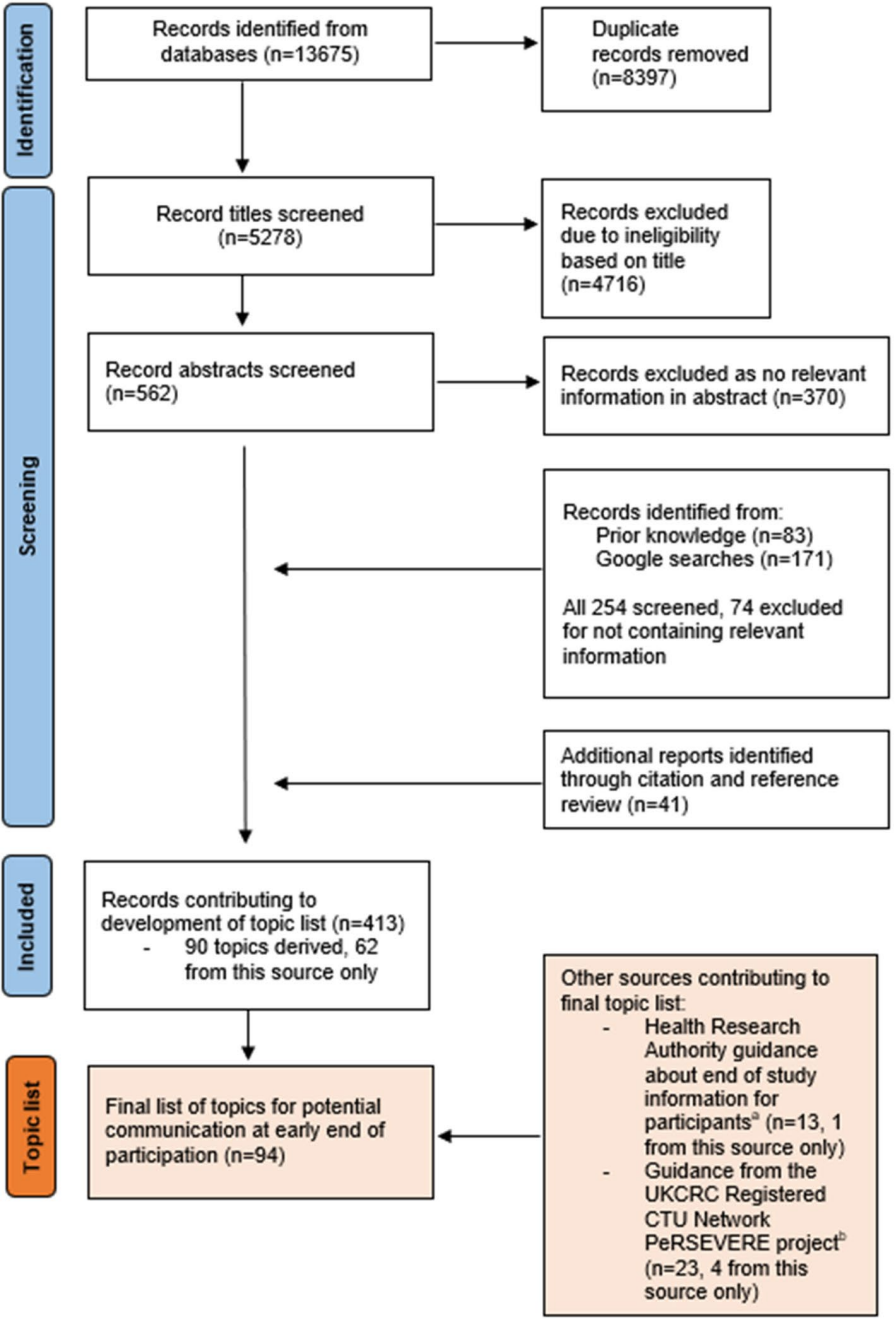
Modified PRISMA-ScR diagram summarising the literature review used to derive material for the project output. ^a^https://s3.eu-west-2.amazonaws.com/www.hra.nhs.uk/media/documents/information-participants-end-study-guidance-researchers.pdf. ^b^https://persevereprinciples.org

The literature review identified 413 relevant results from all sources, of which 26 were ‘key papers’ (see Supplementary information) and 387 were ‘indirectly relevant’. Figure 1 shows the number of records identified, screened and excluded.

The results of the review suggested that the needs of participants who stop taking part early have not been considered much previously. This is reflected in the small number of directly relevant results. The identified reports did not provide enough information on the final two search objectives (about changing information to specifically communicate with early-stopping participants, or about methods of communication) to be able to report any summary regarding these points here. Instead, we explored these points through the group work.

As an additional example showing the scarcity of relevant literature, we found 155 reports recommending that participants receive information about how and when they can receive the results of studies they took part in. Only around 10% of these were clear (or implied) that participants who stopped early should also get this information. A few of the remainder said that these participants had been (or should be) actively excluded from this information sharing, without giving a clear justification [19–21].

The draft topic list was reviewed by 17 research professionals and 4 public contributors, leading to useful refinement. The final topic list contained 94 items to consider when communicating with research participants who stop taking part early (see Supplementary information). Of the 94 items, the project lead classified 25 as general items for any participants ending their participation (whether early or not), 52 as general but with some specific things to say to participants who stop early, and 17 as specific items only relevant to participants who stop early.

### Recruiting public and professional volunteers

Following 31 expressions of interest, a development group (seven contributors) and a review group (15 contributors) were convened, including individuals with diverse characteristics and experiences.

Across both groups, there were individuals ranging from under 30 years old to over 66. There was a roughly even split of individuals identifying themselves as female and male. Most of the contributors identified as White ethnicity, but a few identified as Asian or mixed ethnicity. Contributors were from across the UK and had personal experiences of a range of health conditions. Some contributors identified as neurodivergent. Around half of the development group had personal experience of stopping research participation early. Nearly half of all the expressions of interest were received via the National Institute of Health and Care Research People in Research website [22].

We involved eight research nurses in the project, six of whom were identified via Twitter. All the research nurses inputted into the project in some way, e.g. at least through a short discussion with the project lead about their experiences working with research participants during the process of stopping their participation early.

### Developing the researcher resource

**Fig. 2:**
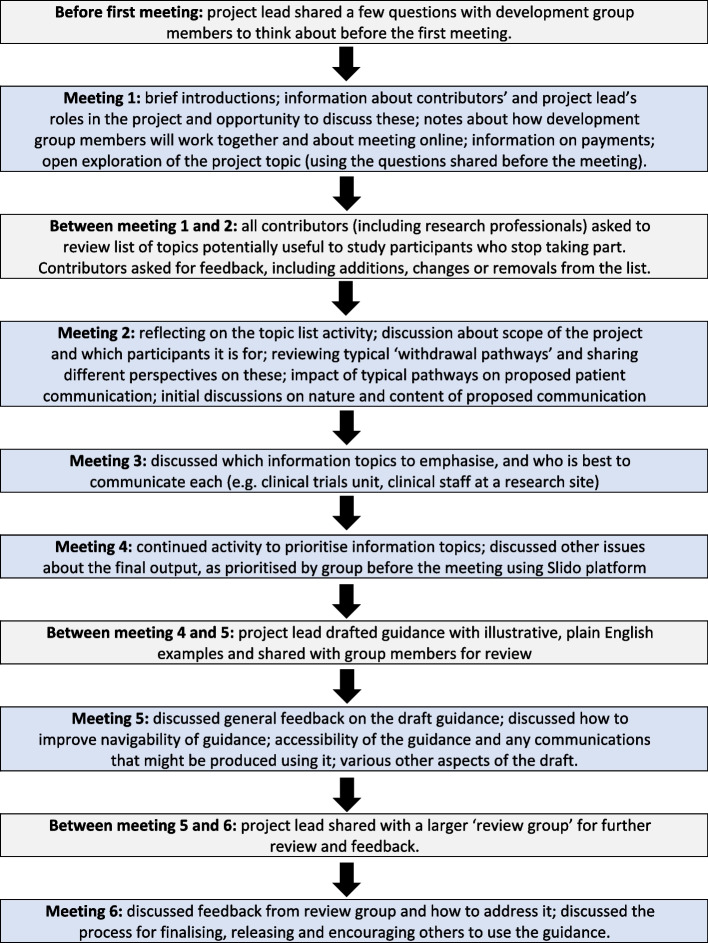
Sequence of patient and public involvement activities and meetings used to develop our online resource

The development group meetings were all held online in 2021–22. The meetings were led and facilitated by the project lead. Most attendees at each meeting were public contributors. Other attendees included other researchers from the CTRU and, at some meetings, involved research nurses. The eventual series of meetings and activities, agreed together as we progressed through the work, is shown in Fig. 2.

We made decisions by informal consensus, following discussion and debate in the group meetings. After each meeting, the project lead shared a summary of the discussion and next steps with all attendees. The project lead took care to check in with individual group members as needed to make sure they were happy with the progress of the project.

Prior to the first development group meeting, attendees were sent questions to consider. These included questions about common reasons for participants to end their involvement in a study, group members’ own experiences of stopping taking part and how that process was for them, how participants feel when going through that event, and what sort of information is useful to participants when they stop taking part. Attendees were offered reassurance that they only needed to share as much about their own experiences as they were comfortable with.

At the first group meeting, these questions formed a loose structure for an open exploration of the topic we were going to discuss together. This helped us understand the issues and where we might have differences of opinion across the group.

We recognised at this point that research participants might stop taking part in a wide range of circumstances and for many different reasons (including where stopping was not actually participants’ choice, and where participants stop without communicating their wishes to researchers). We therefore understood that our guidance may need to account for this.

The original plan had been to develop a template communication for researchers to use. However, it was decided early on that the variation in types of study and participant potentially in scope of the work might make it challenging to make a single template. Instead, we agreed to develop guidance with example wording, and we encourage individual research teams to develop their own templates using these.

We also discussed how participants might have mixed feelings about stopping, including disappointment, relief, guilt, anger or awkwardness about telling the researchers about their decision to stop taking part. We agreed that there would not be a one-size-fits-all approach to providing information to participants, so each individual’s circumstances would need to be considered.

Following the first group meeting, members were asked to review the topic list and provide feedback. This exercise suggested that most of the topic list items were potentially relevant to some participants. We therefore agreed to leave most items in our guidance, rather than trying to define a ‘core information set’ or similar. We would leave it to research teams (including public contributors) to decide what would likely be most relevant to their participants, and to individual participants receiving the communication to decide which information was most relevant to them.

**Fig. 3:**
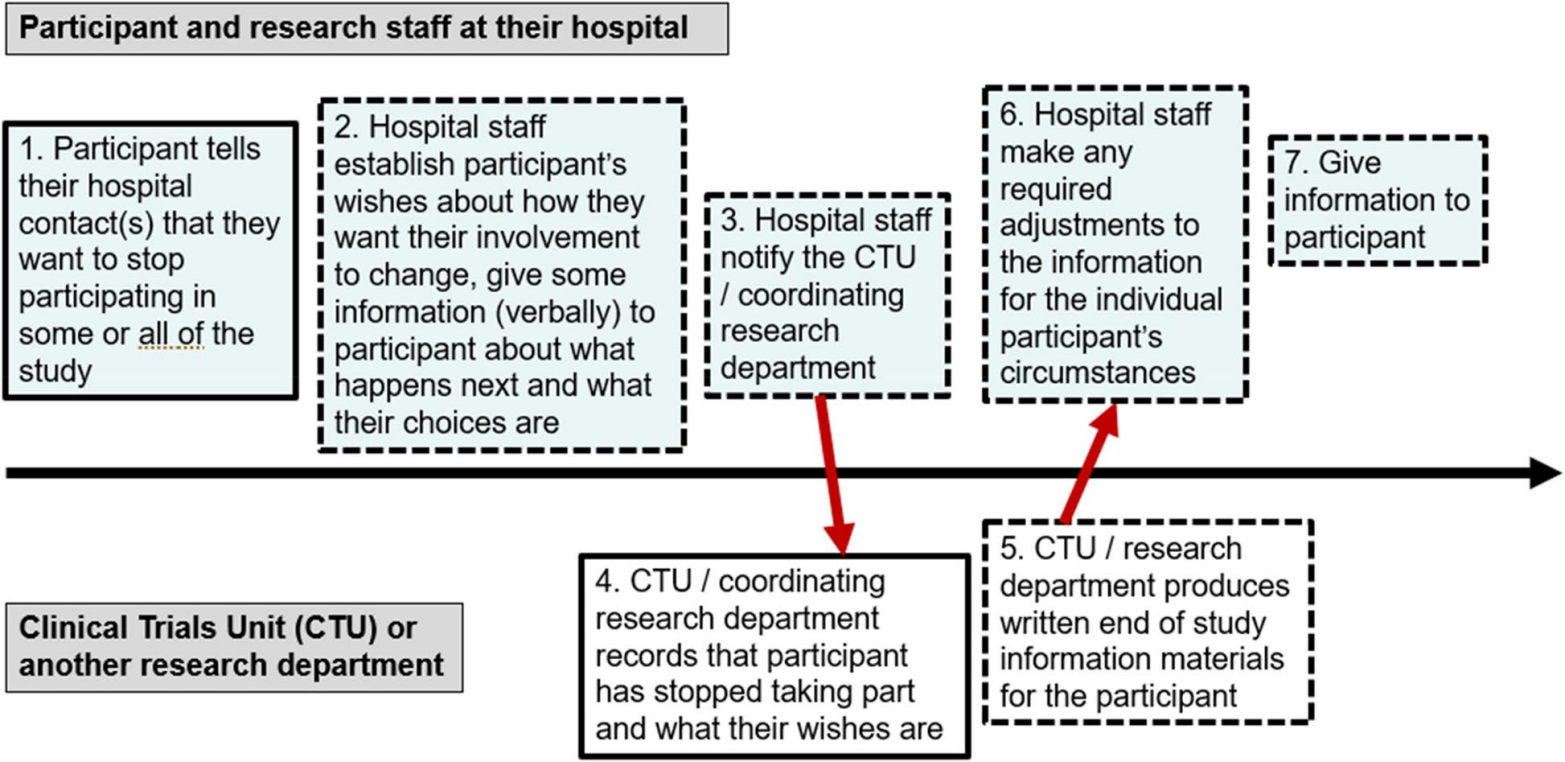
Example pathway for how information could be provided to research participants when they stop participating. Dotted borders indicate where a step may not happen at present, or not universally, or not always in a timely manner (based on our experiences and other, anecdotal evidence). It was important to acknowledge this in our discussions about providing information to participants who stop taking part. Solid borders indicate steps that already happen routinely

At subsequent meetings, we reviewed and discussed typical ‘pathways’ for participants stopping their involvement in a study (see an example in Fig. 3). The examples were based on academically-sponsored healthcare clinical trials in the UK run by clinical trials units such as the CTRU, and summarised who would be notified of a participant’s wishes and when. This was a useful opportunity to share different perspectives on the process. The public contributors were surprised by how long it can sometimes take for a clinical trials unit to find out that a participant has stopped taking part. Understanding the process impacted on the final guidance. For example, if it might not be feasible to get the planned communication to a participant soon after they stop taking part, this might affect what content is useful to include in the communication.

We discussed exactly who the communication would be for. We confirmed that it would be for after a participant had stopped (or said they wanted to stop) taking part, not before. We agreed it was primarily for participants who stop all or most active parts of a study, such as attending hospital visits or completing questionnaires. This would include where stopping was not a participant’s choice. It could also be used in the case where researchers and participants lose contact with one another, though only if researchers considered it appropriate and had enough certainty that the participant’s contact details were still valid. We excluded cases where participants die or lose capacity to consent during a study (in which case the communication would be with their family or carer). We also excluded situations where participants have not stopped taking part but are observed to be disengaging with a study, so may be at risk of stopping.

We discussed various other aspects of the planned communication, including making sure it is accessible, not too long, comes from a trusted source and makes clear if participants might be expected to do something in response to the message.

### Resource review and finalisation

The draft guidance was reviewed by 14 out of 15 of the review group members. Members were asked to give any feedback they wanted. They were also provided with some specific questions to consider about the quality and suitability of the resource. Reviewers were generally supportive of the aims of the work and did not recommend significant alterations to the guidance or the example wording. In response to their feedback about ease of navigation, we agreed to present the guidance primarily in an online format, giving users the chance to engage with it in whatever way suits them best. The full document is also available as a download to those who might want it, along with other downloadable resources.

**Fig. 4:**
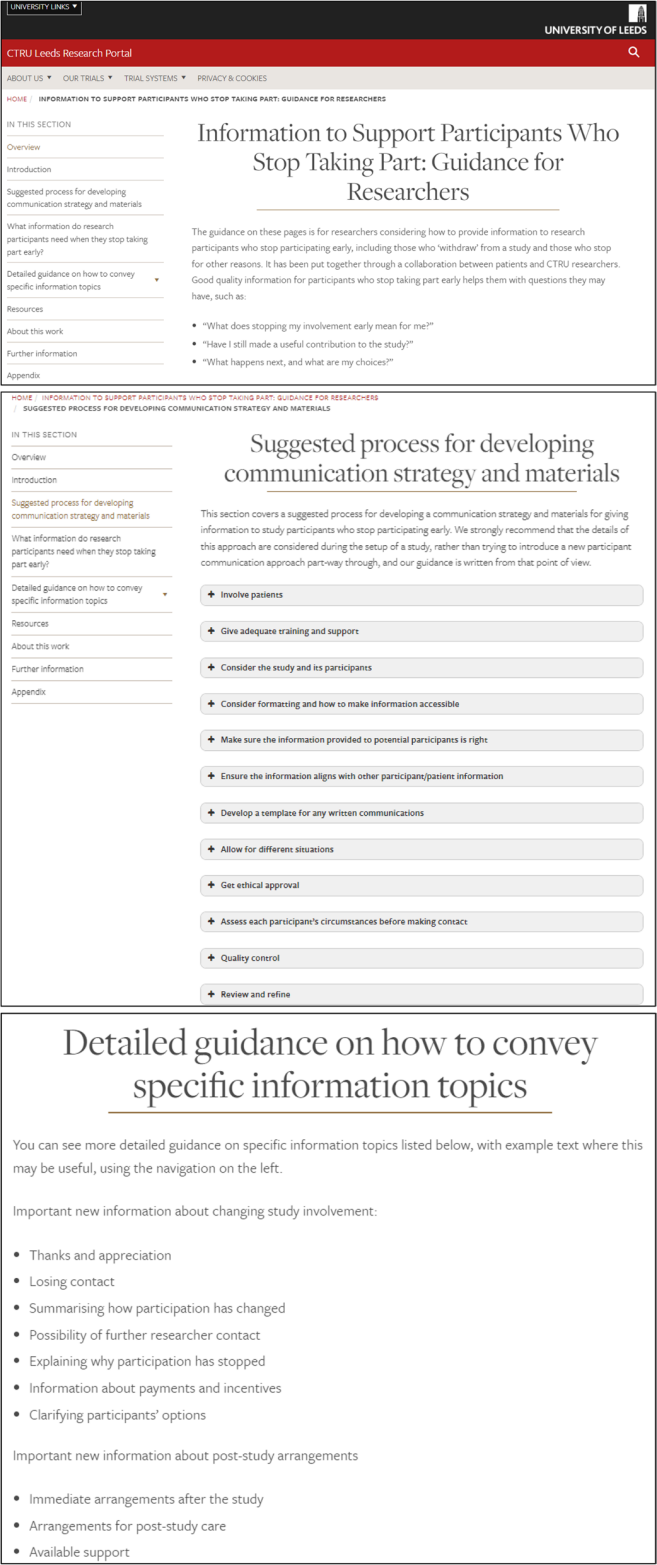
Screenshots of the final online resource. **a** Resource homepage. **b** Suggested process for developing communication strategy and materials. **c** Detailed guidance on how to convey specific topics

The final guidance is available online [23]. Some illustrative screenshots are shown in Fig. 4. We will continue to make amendments to this in future as required, and make additional resources available for download as we develop them.

The guidance includes suggestions for how to introduce this sort of communication to Research Ethics Committees (RECs), highlighting that the guidance was developed with public contributors. Given the potential importance of what participants who stop taking part are told before they agree to take part, we included some novel recommendations on what to include in pre-study information. These suggestions have since been further developed into template wording for pre-study information, available on the Persevere project website [24].

## Discussion

We jointly developed a resource to help researchers think about how to provide useful and sensitively-worded information to research participants who stop taking part. Table 1 shows actions that public contributors and researchers might do in response to this work.

**Table 1:** Suggestions for what public contributors and researchers might do in response to our guidance

**Three things public contributors could do in response to this new resource:**
- Consider your own view. When could providing an information sheet to research participants who stop taking part be helpful and when might it be inappropriate?
- Talk to researchers you work with – have they seen this guidance already? Are they thinking about this issue? Will they consider developing communications for research participants who stop taking part?
- When reviewing research patient information sheets/leaflets, check it seems clear what would happen if participants stop taking part and that there would not be any unwelcome surprises for research participants (or researchers).
**Three things researchers could do in response to this new resource:**
- Consider participants in your research. Might stopping early be particularly difficult for any reason? Might they need more support?
- Consider how to support participants who stop taking part when you are designing your research. If you and public contributors agree it could be appropriate to use this sort of end of participation information sheet in your research, build it in from the start alongside other planned participant communications.
- Use our guidance and the linked Persevere project recommendations to ensure research patient information sheets/leaflets are clear about what will happen if participants stop taking part.

We hope our guidance will encourage everyone involved in research to consider a balanced approach to communicating with this group of participants. While some might not want any further contact, others might. A ‘patient-centred’ approach [25] might mean researchers should find out what each participant wants, wherever possible. Further guidance about how to do this is available on the Persevere website [4].

The literature review reported here confirms that this issue has not been thought about much before. There are even several examples of participants who stopped taking part being specifically excluded from receiving information without a clear justification. This may mean participants are not generally given much information when they stop taking part. This may lead to feelings of ‘abandonment’ or generally a negative impression of research participation, especially if they stopped taking part because of some problem they experienced while taking part.

We acknowledge that we may learn more when our guidance is put into practice. We have made one REC-approved set of documents available in the ‘Resources’ section of our guidance. The REC reviewing those documents had only minor comments to do with making sure participants have access to all the information they might need, and access to different methods of contacting research staff. They also advised about the number of times it would be appropriate to try to recontact individuals who had lost contact with research teams.

If possible, we will continue to add real-life, REC-approved examples to our website for others to borrow and reuse. We encourage others using the resource to share their feedback and experiences with us so that we can continue to improve it.

Although most of us in the group that developed this resource are public contributors, we cannot be sure how acceptable this type of communication will be to participants in practice. In our guidance, we encourage researchers to plan this end of participation communication alongside planning other participant communications. If participants are used to receiving study-related communications as they progress through a study, then perhaps a communication to mark their ending participation would not be unexpected.

The views of REC members are relevant as they are key in approving (or not) the use of this sort of participant communication. Following the release of the resource, we have carried out a survey to learn more about REC members’ views on this sort of communication. We plan to report results of this survey separately.

We suggest that the process we have followed could be a useful way to produce patient-facing material in future, or guidance about patient-facing material. In particular, we would emphasise public contributors and research professionals first having an open discussion about the topic at hand to get a shared understanding, then working through the detail. Relying on informal consensus – addressing concerns until a suitable compromise can be reached – may mean the output is more likely to be acceptable to all stakeholders. The use of a more focussed ‘development group’ and a separate ‘review group’ to check the output has also worked well in our case.

We do not yet have evidence of effects of end-of-participation communications on participants’ experiences in research, and we do not know if it makes it more likely for participants to stay involved in studies with less commitment (rather than stopping all involvement). It would also be useful to understand the cost implications of the additional participant communication. We suggest these would be a useful areas of future study. Researchers at the Leeds CTRU have proposed a ‘study within a trial’ that could address some of these questions [26].

The main strength of our work is that it was co-developed by people with direct lived experience not just of research participation, but of stopping participation early. Our output is broadly applicable to different types of clinical research and can be used flexibly. It nonetheless provides detailed guidance, including suggested wording for researchers to use as a starting point. We have also taken into account the (limited amount of) available literature on this topic. The methods used to develop the ‘topic list’ that fed into the resource development give assurance that the list was comprehensive.

The main limitation is perhaps that, as above, we have not yet tested the guidance in real study settings. Public contributors were not involved in the detail of the literature review supporting this work, but there was patient and public involvement in the initial grant application describing the planned project. Public contributors also reviewed the topic list that was the main output from the literature review. Use of qualitative research methods might have added rigour to our process, but we suggest the deliberative and collaborative nature of this work are its strengths.

When reflecting on the patient and public involvement during preparation of this paper, we discussed the importance of good facilitation in a process like this. A good facilitator communicates clearly, builds trust, is open to change, and is skilled in managing different perspectives to find common ground and compromise.

The fact that the involvement had all taken place online was new for some of us in 2021. Occasionally it posed technical challenges, and meant that activities could not be too long (to avoid ‘screen fatigue’). However, it also allowed for some breaking down of geographical barriers, and meant people could be involved who otherwise would not have been able to.

## Conclusions

We developed this guidance to address an important issue in research, involving a group of participants who may be considered ‘underserved’ by the lack of guidance for researchers so far about how to support them. We encourage others to use our guidance and propose further developments. Better communication with participants who stop taking part might give them a better overall experience of research. It may also improve research quality in various ways, including making it more likely for participants to give feedback that could improve study quality.

### Supplementary Information

**Additional file 1.** PubMed search strategy.

**Additional file 2.** Key papers from scoping literature review.

**Additional file 3.** List of topics or ideas for what might need to be communicated to participants when they stop taking part in a clinical trial or other study.

**Additional file 4.** Short form GRIPP2 reporting checklist.

## Data Availability

The full results of the literature review supporting this work are available online at https://ctru.leeds.ac.uk/information-to-support-participants-who-stop-taking-part/resources/. All other relevant materials are available in or with this article.

## Abbreviations

CINAHL: Cumulative Index to Nursing and Allied Health Literature
CTRU: Clinical Trials Research Unit (University of Leeds)
PPIE: Patient and Public Involvement and Engagement
PRISMA-ScR: Preferred Reporting Items for Systematic reviews and Meta-Analyses extension for Scoping Reviews
REC: Research Ethics Committee
