# Supplementary material for: How can we support research participants who stop taking part? Communications guidance developed through public-researcher collaboration
Source: Res Involv Engagem. 2024 Apr 18;10:39. doi: 10.1186/s40900-024-00572-4 (PMC11025252; doi:10.1186/s40900-024-00572-4)
Supplement: Supplementary file 1 — Additional file 1. PubMed search strategy. [file 40900_2024_572_MOESM1_ESM.docx]

**Supplement 1: PubMed search strategy**

NB this search was composed of multiple smaller searches exploring different concepts, due to the possibility of reports describing relevant content in different ways. We also focussed on finding terms in article titles and not abstracts, because many of the search terms would be present in many abstracts (i.e. low specificity).

All stages of the final search (after initial scoping) are presented below for transparency, with the final, combined search term in its entirety at the bottom.

| **Search number** | **Topics / Combination** | **Search term** |
| --- | --- | --- |
| **1** | **End** | ((((((((((((end[Title]) OR (ends[Title])) OR (ending[Title])) OR (stop*[Title])) OR (finish*[Title])) OR (complet*[Title])) OR (termin*[Title])) OR (fail*[Title])) OR (halt*[Title])) OR (cess*[Title])) OR (ceas*[Title])) OR (clos*[Title])) NOT (((((point*[Title]) OR (life[Title])) OR (stage[Title])) OR (user*[Title])) OR (end result*[Title])) |
| **2** | **Trial** | ((trial*[Title]) OR (stud*[Title])) OR (research*[Title]) |
| **3** | **Information** | (((((((((((((((inform*[Title]) OR (communic*[Title])) OR (message*[Title])) OR (decide*[Title])) OR (decis*[Title])) OR (document*[Title])) OR (feedback*[Title])) OR (survey*[Title])) OR (contact*[Title])) OR (feed back[Title])) OR (fed back[Title])) OR (debrief*[Title])) OR (offer*[Title])) OR (disclos*[Title])) OR (notif*[Title])) OR (disseminat*[Title]) |
| **4** | **1 AND 2 AND 3** | ((((((((((((((end[Title]) OR (ends[Title])) OR (ending[Title])) OR (stop*[Title])) OR (finish*[Title])) OR (complet*[Title])) OR (termin*[Title])) OR (fail*[Title])) OR (halt*[Title])) OR (cess*[Title])) OR (ceas*[Title])) OR (clos*[Title])) NOT (((((point*[Title]) OR (life[Title])) OR (stage[Title])) OR (user*[Title])) OR (end result*[Title])))  **AND** (((trial*[Title]) OR (stud*[Title])) OR (research*[Title])))  **AND** ((((((((((((((((inform*[Title]) OR (communic*[Title])) OR (message*[Title])) OR (decide*[Title])) OR (decis*[Title])) OR (document*[Title])) OR (feedback*[Title])) OR (survey*[Title])) OR (contact*[Title])) OR (feed back[Title])) OR (fed back[Title])) OR (debrief*[Title])) OR (offer*[Title])) OR (disclos*[Title])) OR (notif*[Title])) OR (disseminat*[Title])) |
| **5** | **Withdraw** | (((loss[Title]) OR (lost[Title])) AND (follow[Title])) OR ((((((withdr*[Title]) OR (discontinu*[Title])) OR (dropout*[Title])) OR (drop-out*[Title])) OR (prematur*[Title])) OR (off trial[Title])) |
| **6** | **2 AND 3 AND 5** | ((((trial*[Title]) OR (stud*[Title])) OR (research*[Title]))  **AND** ((((((((((((((((inform*[Title]) OR (communic*[Title])) OR (message*[Title])) OR (decide*[Title])) OR (decis*[Title])) OR (document*[Title])) OR (feedback*[Title])) OR (survey*[Title])) OR (contact*[Title])) OR (feed back[Title])) OR (fed back[Title])) OR (debrief*[Title])) OR (offer*[Title])) OR (disclos*[Title])) OR (notif*[Title])) OR (disseminat*[Title])))  **AND** ((((loss[Title]) OR (lost[Title])) AND (follow[Title])) OR ((((((withdr*[Title]) OR (discontinu*[Title])) OR (dropout*[Title])) OR (drop-out*[Title])) OR (prematur*[Title])) OR (off trial[Title]))) |
| **7** | **Participation** | (((((take part[Title]) OR (taking part[Title])) OR (particip*[Title])) OR (engag*[Title])) OR (subject*[Title])) OR (volunteer*[Title]) |
| **8** | **1 AND 2 AND 7** | ((((((((((((((end[Title]) OR (ends[Title])) OR (ending[Title])) OR (stop*[Title])) OR (finish*[Title])) OR (complet*[Title])) OR (termin*[Title])) OR (fail*[Title])) OR (halt*[Title])) OR (cess*[Title])) OR (ceas*[Title])) OR (clos*[Title])) NOT (((((point*[Title]) OR (life[Title])) OR (stage[Title])) OR (user*[Title])) OR (end result*[Title])))  **AND** (((trial*[Title]) OR (stud*[Title])) OR (research*[Title])))  **AND** ((((((take part[Title]) OR (taking part[Title])) OR (particip*[Title])) OR (engag*[Title])) OR (subject*[Title])) OR (volunteer*[Title])) |
| **9** | **1 AND 5 AND 7** | ((((((((((((((end[Title]) OR (ends[Title])) OR (ending[Title])) OR (stop*[Title])) OR (finish*[Title])) OR (complet*[Title])) OR (termin*[Title])) OR (fail*[Title])) OR (halt*[Title])) OR (cess*[Title])) OR (ceas*[Title])) OR (clos*[Title])) NOT (((((point*[Title]) OR (life[Title])) OR (stage[Title])) OR (user*[Title])) OR (end result*[Title]))) AND ((((loss[Title]) OR (lost[Title]))  **AND** (follow[Title])) OR ((((((withdr*[Title]) OR (discontinu*[Title])) OR (dropout*[Title])) OR (drop-out*[Title])) OR (prematur*[Title])) OR (off trial[Title]))))  **AND** ((((((take part[Title]) OR (taking part[Title])) OR (particip*[Title])) OR (engag*[Title])) OR (subject*[Title])) OR (volunteer*[Title])) |
| **10** | **Continuous consent** | ((((((continu*[Title]) OR (on-going[Title])) OR (ongoing[Title])) OR (dynamic[Title])) OR (maintain*[Title])) OR (mainten*[Title])) AND (consent*[Title]) |
| **11** | **2 AND 10** | (((trial*[Title]) OR (stud*[Title])) OR (research*[Title]))  **AND** (((((((continu*[Title]) OR (on-going[Title])) OR (ongoing[Title])) OR (dynamic[Title])) OR (maintain*[Title])) OR (mainten*[Title])) AND (consent*[Title])) |
| **12** | **2 AND 3 AND 7** | ((((trial*[Title]) OR (stud*[Title])) OR (research*[Title]))  **AND** ((((((((((((((((inform*[Title]) OR (communic*[Title])) OR (message*[Title])) OR (decide*[Title])) OR (decis*[Title])) OR (document*[Title])) OR (feedback*[Title])) OR (survey*[Title])) OR (contact*[Title])) OR (feed back[Title])) OR (fed back[Title])) OR (debrief*[Title])) OR (offer*[Title])) OR (disclos*[Title])) OR (notif*[Title])) OR (disseminat*[Title])))  **AND** ((((((take part[Title]) OR (taking part[Title])) OR (particip*[Title])) OR (engag*[Title])) OR (subject*[Title])) OR (volunteer*[Title])) |
| **13** | **Obligations** | (((oblig*[Title]) OR (duty[Title])) OR (duties[Title])) OR (require*[Title]) |
| **14** | **1 AND 2 AND 13** | ((((((((((((((end[Title]) OR (ends[Title])) OR (ending[Title])) OR (stop*[Title])) OR (finish*[Title])) OR (complet*[Title])) OR (termin*[Title])) OR (fail*[Title])) OR (halt*[Title])) OR (cess*[Title])) OR (ceas*[Title])) OR (clos*[Title])) NOT (((((point*[Title]) OR (life[Title])) OR (stage[Title])) OR (user*[Title])) OR (end result*[Title])))  **AND** (((trial*[Title]) OR (stud*[Title])) OR (research*[Title])))  **AND** ((((oblig*[Title]) OR (duty[Title])) OR (duties[Title])) OR (require*[Title])) |
| **15** | **Information needs** | information need*[Title] |
| **16** | **2 AND 15** | (((trial*[Title]) OR (stud*[Title])) OR (research*[Title]))  **AND** (information need*[Title]) |
| **17** | **4 OR 6 OR 8 OR 9 OR 11 OR 12 OR 14 OR 16** | (((((((((((((((((((((end[Title]) OR (ends[Title])) OR (ending[Title])) OR (stop*[Title])) OR (finish*[Title])) OR (complet*[Title])) OR (termin*[Title])) OR (fail*[Title])) OR (halt*[Title])) OR (cess*[Title])) OR (ceas*[Title])) OR (clos*[Title])) NOT (((((point*[Title]) OR (life[Title])) OR (stage[Title])) OR (user*[Title])) OR (end result*[Title]))) AND (((trial*[Title]) OR (stud*[Title])) OR (research*[Title]))) AND ((((((((((((((((inform*[Title]) OR (communic*[Title])) OR (message*[Title])) OR (decide*[Title])) OR (decis*[Title])) OR (document*[Title])) OR (feedback*[Title])) OR (survey*[Title])) OR (contact*[Title])) OR (feed back[Title])) OR (fed back[Title])) OR (debrief*[Title])) OR (offer*[Title])) OR (disclos*[Title])) OR (notif*[Title])) OR (disseminat*[Title])))  **OR** (((((trial*[Title]) OR (stud*[Title])) OR (research*[Title])) AND ((((((((((((((((inform*[Title]) OR (communic*[Title])) OR (message*[Title])) OR (decide*[Title])) OR (decis*[Title])) OR (document*[Title])) OR (feedback*[Title])) OR (survey*[Title])) OR (contact*[Title])) OR (feed back[Title])) OR (fed back[Title])) OR (debrief*[Title])) OR (offer*[Title])) OR (disclos*[Title])) OR (notif*[Title])) OR (disseminat*[Title]))) AND ((((loss[Title]) OR (lost[Title])) AND (follow[Title])) OR ((((((withdr*[Title]) OR (discontinu*[Title])) OR (dropout*[Title])) OR (drop-out*[Title])) OR (prematur*[Title])) OR (off trial[Title])))))  **OR** (((((((((((((((end[Title]) OR (ends[Title])) OR (ending[Title])) OR (stop*[Title])) OR (finish*[Title])) OR (complet*[Title])) OR (termin*[Title])) OR (fail*[Title])) OR (halt*[Title])) OR (cess*[Title])) OR (ceas*[Title])) OR (clos*[Title])) NOT (((((point*[Title]) OR (life[Title])) OR (stage[Title])) OR (user*[Title])) OR (end result*[Title]))) AND (((trial*[Title]) OR (stud*[Title])) OR (research*[Title]))) AND ((((((take part[Title]) OR (taking part[Title])) OR (particip*[Title])) OR (engag*[Title])) OR (subject*[Title])) OR (volunteer*[Title]))))  **OR** (((((((((((((((end[Title]) OR (ends[Title])) OR (ending[Title])) OR (stop*[Title])) OR (finish*[Title])) OR (complet*[Title])) OR (termin*[Title])) OR (fail*[Title])) OR (halt*[Title])) OR (cess*[Title])) OR (ceas*[Title])) OR (clos*[Title])) NOT (((((point*[Title]) OR (life[Title])) OR (stage[Title])) OR (user*[Title])) OR (end result*[Title]))) AND ((((loss[Title]) OR (lost[Title])) AND (follow[Title])) OR ((((((withdr*[Title]) OR (discontinu*[Title])) OR (dropout*[Title])) OR (drop-out*[Title])) OR (prematur*[Title])) OR (off trial[Title])))) AND ((((((take part[Title]) OR (taking part[Title])) OR (particip*[Title])) OR (engag*[Title])) OR (subject*[Title])) OR (volunteer*[Title]))))  **OR** ((((trial*[Title]) OR (stud*[Title])) OR (research*[Title])) AND (((((((continu*[Title]) OR (on-going[Title])) OR (ongoing[Title])) OR (dynamic[Title])) OR (maintain*[Title])) OR (mainten*[Title])) AND (consent*[Title]))))  **OR** (((((trial*[Title]) OR (stud*[Title])) OR (research*[Title])) AND ((((((((((((((((inform*[Title]) OR (communic*[Title])) OR (message*[Title])) OR (decide*[Title])) OR (decis*[Title])) OR (document*[Title])) OR (feedback*[Title])) OR (survey*[Title])) OR (contact*[Title])) OR (feed back[Title])) OR (fed back[Title])) OR (debrief*[Title])) OR (offer*[Title])) OR (disclos*[Title])) OR (notif*[Title])) OR (disseminat*[Title]))) AND ((((((take part[Title]) OR (taking part[Title])) OR (particip*[Title])) OR (engag*[Title])) OR (subject*[Title])) OR (volunteer*[Title]))))  **OR** (((((((((((((((end[Title]) OR (ends[Title])) OR (ending[Title])) OR (stop*[Title])) OR (finish*[Title])) OR (complet*[Title])) OR (termin*[Title])) OR (fail*[Title])) OR (halt*[Title])) OR (cess*[Title])) OR (ceas*[Title])) OR (clos*[Title])) NOT (((((point*[Title]) OR (life[Title])) OR (stage[Title])) OR (user*[Title])) OR (end result*[Title]))) AND (((trial*[Title]) OR (stud*[Title])) OR (research*[Title]))) AND ((((oblig*[Title]) OR (duty[Title])) OR (duties[Title])) OR (require*[Title]))))  **OR** ((((trial*[Title]) OR (stud*[Title])) OR (research*[Title])) AND (information need*[Title])) |
| **18** | **MeSH terms** | ("Patient Dropouts"[MAJR]) AND ("Informed Consent"[MAJR]) |
| **19 (Final search term as entered)** | **17 OR 18** | ((((((((((((((((((((((end[Title]) OR (ends[Title])) OR (ending[Title])) OR (stop*[Title])) OR (finish*[Title])) OR (complet*[Title])) OR (termin*[Title])) OR (fail*[Title])) OR (halt*[Title])) OR (cess*[Title])) OR (ceas*[Title])) OR (clos*[Title])) NOT (((((point*[Title]) OR (life[Title])) OR (stage[Title])) OR (user*[Title])) OR (end result*[Title]))) AND (((trial*[Title]) OR (stud*[Title])) OR (research*[Title]))) AND ((((((((((((((((inform*[Title]) OR (communic*[Title])) OR (message*[Title])) OR (decide*[Title])) OR (decis*[Title])) OR (document*[Title])) OR (feedback*[Title])) OR (survey*[Title])) OR (contact*[Title])) OR (feed back[Title])) OR (fed back[Title])) OR (debrief*[Title])) OR (offer*[Title])) OR (disclos*[Title])) OR (notif*[Title])) OR (disseminat*[Title]))) OR (((((trial*[Title]) OR (stud*[Title])) OR (research*[Title])) AND ((((((((((((((((inform*[Title]) OR (communic*[Title])) OR (message*[Title])) OR (decide*[Title])) OR (decis*[Title])) OR (document*[Title])) OR (feedback*[Title])) OR (survey*[Title])) OR (contact*[Title])) OR (feed back[Title])) OR (fed back[Title])) OR (debrief*[Title])) OR (offer*[Title])) OR (disclos*[Title])) OR (notif*[Title])) OR (disseminat*[Title]))) AND ((((loss[Title]) OR (lost[Title])) AND (follow[Title])) OR ((((((withdr*[Title]) OR (discontinu*[Title])) OR (dropout*[Title])) OR (drop-out*[Title])) OR (prematur*[Title])) OR (off trial[Title]))))) OR (((((((((((((((end[Title]) OR (ends[Title])) OR (ending[Title])) OR (stop*[Title])) OR (finish*[Title])) OR (complet*[Title])) OR (termin*[Title])) OR (fail*[Title])) OR (halt*[Title])) OR (cess*[Title])) OR (ceas*[Title])) OR (clos*[Title])) NOT (((((point*[Title]) OR (life[Title])) OR (stage[Title])) OR (user*[Title])) OR (end result*[Title]))) AND (((trial*[Title]) OR (stud*[Title])) OR (research*[Title]))) AND ((((((take part[Title]) OR (taking part[Title])) OR (particip*[Title])) OR (engag*[Title])) OR (subject*[Title])) OR (volunteer*[Title])))) OR (((((((((((((((end[Title]) OR (ends[Title])) OR (ending[Title])) OR (stop*[Title])) OR (finish*[Title])) OR (complet*[Title])) OR (termin*[Title])) OR (fail*[Title])) OR (halt*[Title])) OR (cess*[Title])) OR (ceas*[Title])) OR (clos*[Title])) NOT (((((point*[Title]) OR (life[Title])) OR (stage[Title])) OR (user*[Title])) OR (end result*[Title]))) AND ((((loss[Title]) OR (lost[Title])) AND (follow[Title])) OR ((((((withdr*[Title]) OR (discontinu*[Title])) OR (dropout*[Title])) OR (drop-out*[Title])) OR (prematur*[Title])) OR (off trial[Title])))) AND ((((((take part[Title]) OR (taking part[Title])) OR (particip*[Title])) OR (engag*[Title])) OR (subject*[Title])) OR (volunteer*[Title])))) OR ((((trial*[Title]) OR (stud*[Title])) OR (research*[Title])) AND (((((((continu*[Title]) OR (on-going[Title])) OR (ongoing[Title])) OR (dynamic[Title])) OR (maintain*[Title])) OR (mainten*[Title])) AND (consent*[Title])))) OR (((((trial*[Title]) OR (stud*[Title])) OR (research*[Title])) AND ((((((((((((((((inform*[Title]) OR (communic*[Title])) OR (message*[Title])) OR (decide*[Title])) OR (decis*[Title])) OR (document*[Title])) OR (feedback*[Title])) OR (survey*[Title])) OR (contact*[Title])) OR (feed back[Title])) OR (fed back[Title])) OR (debrief*[Title])) OR (offer*[Title])) OR (disclos*[Title])) OR (notif*[Title])) OR (disseminat*[Title]))) AND ((((((take part[Title]) OR (taking part[Title])) OR (particip*[Title])) OR (engag*[Title])) OR (subject*[Title])) OR (volunteer*[Title])))) OR (((((((((((((((end[Title]) OR (ends[Title])) OR (ending[Title])) OR (stop*[Title])) OR (finish*[Title])) OR (complet*[Title])) OR (termin*[Title])) OR (fail*[Title])) OR (halt*[Title])) OR (cess*[Title])) OR (ceas*[Title])) OR (clos*[Title])) NOT (((((point*[Title]) OR (life[Title])) OR (stage[Title])) OR (user*[Title])) OR (end result*[Title]))) AND (((trial*[Title]) OR (stud*[Title])) OR (research*[Title]))) AND ((((oblig*[Title]) OR (duty[Title])) OR (duties[Title])) OR (require*[Title])))) OR ((((trial*[Title]) OR (stud*[Title])) OR (research*[Title])) AND (information need*[Title]))) OR (("Patient Dropouts"[MAJR]) AND ("Informed Consent"[MAJR])) |
