# Supplementary material for: How can we support research participants who stop taking part? Communications guidance developed through public-researcher collaboration
Source: Res Involv Engagem. 2024 Apr 18;10:39. doi: 10.1186/s40900-024-00572-4 (PMC11025252; doi:10.1186/s40900-024-00572-4)
Supplement: Supplementary file 2 — Additional file 2. Key papers from scoping literature review. [file 40900_2024_572_MOESM2_ESM.docx]

**Supplement 2: “Key Results” from Scoping Literature Review**

In our review of existing evidence about communication with research participants who stop taking part, we divided results into:

- Key results that were directly relevant to our topic (e.g. if they were about communication with research participant who stop taking part, or more generally about participants’ experiences of stopping taking part [and therefore with content about their information needs], or about information to share with participants at the end of their time taking part [and therefore with content that could be applied to participants who stop taking part early])
- Indirectly-related results that nonetheless contained relevant information for our search, or could be used as ideas for our list of topics to communicate to participants who stop taking part (see main article).

The list of 26 key results is presented below.

| **Source** | **Title** | **Citation** | **Category E.g. end of trial results, health results disclosure, continuous consent etc** | **Topics or considerations for end of study information** | **Setting/ context** |
| --- | --- | --- | --- | --- | --- |
| Database searches | A method to reduce loss to follow-up in clinical trials: Informed, withdrawal of consent. | Cleland JG, Torp-Pedersen C, Coletta AP, Lammiman MJ. A method to reduce loss to follow-up in clinical trials: informed, withdrawal of consent. Eur J Heart Fail. 2004 Jan;6(1):1-2. doi: 10.1016/j.eheart.2003.12.001. PMID: 15012911. | Early stopping process/ information | Withdrawal form for participants to complete | Opinion article, UK |
| Database searches | Emergent biopsychosocial challenges of novel drug development and clinical trial participation: Stopping an experimental modulator drug in order to conceive. | Journal of Cystic Fibrosis. Conference: 41st European Cystic Fibrosis Conference, ECFS 2018. Serbia. 17 (Supplement 3) (pp S13), 2018. | Experiences of stopping early | Counselling/support options to help deal with early stopping | Cystic fibrosis clinical trial, UK |
| Database searches | Improving Information Exchange with Clinical Trial Participants: A Proposal for Industry. | Dietrich J, Alivojvodic J, Seliverstov I, Metcalf M, Jakee K. Improving Information Exchange with Clinical Trial Participants: A Proposal for Industry. Ther Innov Regul Sci. 2017 Sep;51(5):542-550. doi: 10.1177/2168479017725109. PMID: 30231690. | Participants' information needs | How to stay in touch with the research team How to access health data from trial How to contact other trial participants  Patient support group details Individual results Status of trial (recruitment etc)  Support services available  General trial results  Name of drug when approved  Treatment allocation  Other trials  Thank you message  Post-trial care  Feedback  What to know before trying other treatments  More information about treatment received | International survey and focus groups |
| Database searches | Subjects' views of obligations to ensure post-trial access to drugs, care and information: qualitative results from the Experiences of Participants in Clinical Trials (EPIC) study. | Sofaer N, Thiessen C, Goold SD, Ballou J, Getz KA, Koski G, Krueger RA, Weissman JS. Subjects' views of obligations to ensure post-trial access to drugs, care and information: qualitative results from the Experiences of Participants in Clinical Trials (EPIC) study. J Med Ethics. 2009 Mar;35(3):183-8. doi: 10.1136/jme.2008.024711. PMID: 19251971; PMCID: PMC3044680. | Post-study obligations | Information on post trial access to intervention Trial results  Individual results Info on post-trial care Info on possible further adverse events and care for those Info on whether/how further info on adverse events would be made available (re Vioxx) Treatment allocation  [Reminder of] name and dosage of drugs taken Date trial drug would be marketed | Survey of former trial participants in US - depression, arthritis, diabetes |
| Database searches | The ethics of withdrawal from study participation | Gordon EJ, Prohaska TR. The ethics of withdrawal from study participation. Account Res. 2006 Oct-Dec;13(4):285-309. doi: 10.1080/08989620600848645. PMID: 17849641; PMCID: PMC9527709. | Early stopping process/information | Post-stopping medical monitoring required Medical consequences of stopping trial  End of study visits/tests required  Possibility of further contributions to the study  Who to contact (to tell them your intentions / for more info)  Info on returning trial drug/materials  Withdrawal form ('revocation form')  Reasons for any post-'withdrawal' procedures | IRB-approved active studies approved by one Midwestern, academic hospital's IRB between 1992 and November, 2002 (114 consent forms reviewed as sample) |
| Database searches | Understanding what information is valued by research participants, and why. | Wilkins CH, Mapes BM, Jerome RN, Villalta-Gil V, Pulley JM, Harris PA. Understanding What Information Is Valued By Research Participants, And Why. Health Aff (Millwood). 2019 Mar;38(3):399-407. doi: 10.1377/hlthaff.2018.05046. PMID: 30830824; PMCID: PMC6706772. | Information at end of study | Individual genetic results Aggregate results  Treatment allocation  Information about other research participation opportunities  Reminder of data protection info  Info about incentives  How to connect with 'people like me' in the study | Survey of research participants, US |
| Database searches | What does 'respect for persons' require? Attitudes and reported practices of genetics researchers in informing research participants about research. | Miller FA, Hayeems RZ, Li L, Bytautas JP. What does 'respect for persons' require? Attitudes and reported practices of genetics researchers in informing research participants about research. J Med Ethics. 2012 Jan;38(1):48-52. doi: 10.1136/jme.2010.041350. Epub 2011 Jun 17. PMID: 21685149. | Information at end of study | End of study results Individual results  New results from other studies  Funding news  Opportunities to take part in other research  Availability of other health services/support | Survey of researchers' views in setting of cystic fibrosis and autism research (international) |
| Database searches | What information should be fed back to trial participants?-Findings from a Q-methodology study with trial stakeholders. | Trials. Conference: 5th International Clinical Trials Methodology Conference, ICTMC 2019. United Kingdom. 20 (Supplement 1), 2019. | Information at end of study | Trial identifier & full title Study results  Sponsor details  Conflict of interest information  Reminder about practical/scientific info of trial  Update on progress of trial/future plans  Who to contact for more info  Where to find more info  Individual results  Treatment allocation  Thank you message  Patient and public involvement in trial  Date of message  Information about other relevant trials (for information / for potential participation) | Q-methodology exercise with various stakeholders |
| Already known or identified ad hoc during the search period | What happens when a clinical trial is completed? [Online information] | <https://guides.clarahealth.com/what-happens-after-a-clinical-trial-ends/> | Information at end of study | What happens next Information about accessing treatment after end of study How to access trial results Existence of exit interview (/giving feedback in general) Tips on how to adjust 'back to regular schedule' Trial results (what they are) How to keep in touch  Other/future studies How to monitor your health after trial participation Suggestion to thank those who helped you during trial participation (friends, researchers) How to connect with other patients | USA |
| Already known or identified ad hoc during the search period | Debrief sheet [example from a clinical trial run from the Leeds Clinical Trials Research Unit] | N/a | Information at end of study | Message of thanks Statement about importance of your contribution Reminder about purpose of the trial Treatment allocation (when previously blinded) Info on how to interpret knowledge of treatment allocation FAQs (in this case about implications for subsequent treatment) Info on when results of study will be ready Reassurance that if you were on placebo you have done an important thing by taking part | UK clinical trial related to bowel health |
| Already known or identified ad hoc during the search period | A randomised controlled trial of nurse-managed trial conclusion following early phase cancer trial participation | Cox K, Wilson E, Arthur A, Elkan R, Armstrong S. A randomised controlled trial of nurse-managed trial conclusion following early phase cancer trial participation. Br J Cancer. 2005 Jul 11;93(1):41-5. doi: 10.1038/sj.bjc.6602675. PMID: 15986032; PMCID: PMC2361479. | End of study transition | Exit interviews including debriefing ('of decision around completion/withdrawal') and explanation of further follow-up support  Information leaflet: thank you; latest information about drug being tested; news about other participants; details about participant's contribution to cancer research; available support after trial participation; details on further follow-up  Telephone follow-up at 2 weeks post trial: enquiry on general health; identification of unmet info needs; emotional support  Reasons for trial conclusion | Study within early phase cancer trials at two centres in UK |
| Already known or identified ad hoc during the search period | Enhancing cancer trial management: An intervention study of the impact of providing information, trial results and support to patients in phase I and II anti-cancer drug trials at trial conclusion | Eleanor Wilson, Karen Cox, Ruth Elkan, Enhancing cancer trial management: An intervention study of the impact of providing information, trial results and support to patients in phase I and II anti-cancer drug trials at trial conclusion. Clinical Effectiveness in Nursing, Volume 9, Issues 3–4, 2005, 119-132. https://doi.org/10.1016/j.cein.2006.06.003. | End of study transition | Reassurance that what they took part in was worthwhile  Feedback about trial results/status What happens next [generally] Recognition of current feelings/challenges (e.g. disappointment if treatment didn't work; relief; anxiety about illness) Information about support  Feedback about the trial Confirmation that participant made a contribution  How others in the trial fared (e.g. in terms of side effects or health status) Whether trial drug considered a success Whether or not drug will be available in hospitals Availability of support for talking about individual experiences/side effects etc Opportunities for contacting other participants Info on follow-up arrangements Opportunities to give feedback about the trial Recognition that stopping the trial might be an 'adjustment' What to expect in terms of health/side effects and how to manage this More 'personal' info e.g. what it all means for the participant Who to contact in case of questions | Early phase cancer trials |
| From citation & reference reviews | Post-trial obligations in the Declaration of Helsinki 2013: classification, reconstruction and interpretation. | Mastroleo I. Post-trial obligations in the Declaration of Helsinki 2013: classification, reconstruction and interpretation. Dev World Bioeth. 2016 Aug;16(2):80-90. doi: 10.1111/dewb.12099. Epub 2015 Oct 19. PMID: 26481322. | Post-study obligations | Post-trial access to trial treatment Post-trial treatment generally Post-trial access 'to information' [means: trial results, individual results, access to up-to-date info on side effects] Reminder of what group randomised to Withdrawal of the drug from the market for safety reasons | Discussion on ethics, mainly around Declaration of Helsinki and evolution thereof |
| From citation & reference reviews | A qualitative assessment of the experience of participating in a cancer-related clinical trial. | Wootten AC, Abbott JM, Siddons HM, Rosenthal MA, Costello AJ. A qualitative assessment of the experience of participating in a cancer-related clinical trial. Support Care Cancer. 2011 Jan;19(1):49-55. doi: 10.1007/s00520-009-0787-z. Epub 2009 Dec 4. PMID: 19960208. | End of study transition | Recognition of difficult feelings (abandonment, isolation, disappointment if no personal improvement, letting down the trial if stop early) Difficult feelings in family members Uncertainty about the future What help available Help interpreting medical test results Information on referrals | Focus groups, cancer trial participants in Australia (all 3 phases of trials) |
| From citation & reference reviews | Data withdrawal in randomized controlled trials: Defining the problem and proposing solutions: A commentary | Ye C, Giangregorio L, Holbrook A, Pullenayegum E, Goldsmith CH, Thabane L. Data withdrawal in randomized controlled trials: Defining the problem and proposing solutions: a commentary. Contemp Clin Trials. 2011 May;32(3):318-22. doi: 10.1016/j.cct.2011.01.016. Epub 2011 Feb 4. PMID: 21300179. | Data retention | Importance of continued participation Possibility of alternatives that allow continued participation Requesting information on reasons for stopping Willingness to continue data collection (all data/outcome data only) | Canada; literature review of international guidance around data withdrawal |
| From citation & reference reviews | Patient experiences of participation in a radical thoracic surgical trial: findings from the Mesothelioma and Radical Surgery Trial 2 (MARS 2) | Warnock C, Lord K, Taylor B, Tod A. Patient experiences of participation in a radical thoracic surgical trial: findings from the Mesothelioma and Radical Surgery Trial 2 (MARS 2). Trials. 2019 Oct 18;20(1):598. doi: 10.1186/s13063-019-3692-x. PMID: 31627746; PMCID: PMC6798336. | Information at end of study | Post-trial plans including what will happen after the trial Who will be responsible for their ongoing treatment Who to contact for advice Future treatments that might be needed or available Recognition of feelings of loss/abandonment at end of trial Information on the plan and support post-trial Existence of 'care navigator' role to help with post-trial support Exit consultation to cover future treatment and surveillance plans Existence of other support resources | Qualitative interviews, mesothelioma trial, UK |
| Web searches | Withdrawing from a Research Study | https://hrpo.wustl.edu/participants/withdrawing-from-a-study/ | Early stopping process/information | Reminder of right to withdraw without giving reason Why providing reason is useful How to safely stop medications Who to contact in case of further issues Existence of final visit if needed Participants' wishes about further data collection Existence of 'withdrawal form' or similar Situation where further contact might be made by researchers (e.g. for safety reasons) Confirmation about further data collection or not Reminder that existing data will be kept | University guidance, USA |
| Web searches | Debriefing Process Guidance | https://www.umass.edu/research/guidance/debriefing-process-guidance | Debriefing/deception | Availability of debriefing Disclosure of any deception used Why deception used Opportunity to withdraw altogether (including data) from study after knowing full details Contact details Trial results Links to further information Support if upset Reminder of purpose and details of study Further opportunity to ask questions Thank you message Reminder that all data will remain confidential Restrictions (requested) on talking to other participants | University guidance, USA |
| Web searches | Deception and Debriefing Instructions and Sample Form Template | http://provost.umw.edu/irb/files/2019/01/DEBRIEFING-INSTRUCTIONSSAMPLE-2019.pdf | Debriefing/deception | Information about deception Information/reminder about study purpose and methods Option to refuse consent after learning about deception Contact information Information about conselling/support available Information about how data used/retained Trial results Additional resources about research topic Incentive information What happens in case of new risks later on What to do if experience side-effects or negative impact (/who to contact) Independent contact in case of complaints | University guidance, USA |
| Web searches | Exemplar Withdrawal Form | https://www.cardiffmet.ac.uk/research/Documents/Ethics/withdrawal_form_v1_Sept19.docx | Early stopping process/information | Reminder of right to withdraw Possibility or limitation on data destruction | Withdrawal form from UK University |
| Web searches | Participant Information Sheet/Consent Form (template) | https://www.nhmrc.gov.au/sites/default/files/documents/attachments/PICF/PICF-health-social-science-self.doc | End of study transition | Existence of specific processes around stopping early ('special requirements' - also withdrawal of consent form) What has stopped (e.g. data collection) What happens to existing data Trial results Clear title on communications Name and details of study Reminder of rights regarding withdrawal | Research funder guidance, Australia |
| Web searches | Data Management Resources | https://www.nrgoncology.org/Clinical-Trials/Clinical-Trial-Resources/Statistical-and-Data-Management-Center-SDMC-/Data-Management-Resources | Lost contact | Notification that participant has been hard to reach Contact details Encouragement to get in touch Facilitator to get in touch e.g. stamped addressed envelope Reinforce participant's importance in the study Options for other follow-up | Non-profit research organisation, USA |
| Web searches | Thanking Participants | https://ctontario.ca/resources/participant-experience-toolkit/thanking-participants/ | Thank you message/appreciation | Thank you message Title of study Possibility of translating information Personalisation e.g. clinician signature Individual details of participation e.g. how many visits attended, how many samples provided Reminder of purpose of trial Sponsor information  Investigator information Name of site Study website link  Contact information Trial results | Research organisation guidance, Canada |
| Web searches | Debriefing Statements: | https://irb.lafayette.edu/wp-content/uploads/sites/214/2018/04/Debriefing-Statements.pdf | Debriefing/deception | Debriefing when deception used Study title Name of main investigator(s)  Purpose and methods of study  Opportunity to withdraw consent once debriefed Trial results  Contact details  Available support  References for more on the study topic  Thank you message | University guidance, USA |
| Web searches | Feedback and appreciation samples | https://uwaterloo.ca/research/office-research-ethics/research-human-participants/application-process/samples-and-other-supporting-materials/feedback-and-appreciation-samples | Thank you message/appreciation | Thank you message Study title  Reminder of purpose of study  Anticipated contribution of study to the problem  Reminder of ethical review  Contact details  Reminder that data kept confidential Trial results  Name of investigator(s) What happens to data  Reminder that data will be kept securely  Links to other published studies of interest | University guidance, Canada |
| Web searches | “Thank You” Letter at Participant Completion of Clinical Study | http://www.transceleratebiopharmainc.com/wp-content/uploads/2018/04/Thank-you-Letter_Participant-Completion_FINAL.pdf | Thank you message/appreciation | Participant name Thank you message  Total number of people in the trial  Reminder of purpose of study  Overall study status  Timelines for study completion  Acknowledgement of contribution/commitment  Gains hoped for from study  Invitation to join mailing list  Trial results  Link to registry page  Signature/name of who letter is from | International pharma/academia collaboration for improving trials |
