# Supplementary material for: How can we support research participants who stop taking part? Communications guidance developed through public-researcher collaboration
Source: Res Involv Engagem. 2024 Apr 18;10:39. doi: 10.1186/s40900-024-00572-4 (PMC11025252; doi:10.1186/s40900-024-00572-4)
Supplement: Supplementary file 3 — Additional file 3. List of topics or ideas for what might need to be communicated to participants when they stop taking part in a clinical trial or other study. [file 40900_2024_572_MOESM3_ESM.docx]

**Supplement 3: list of topics or ideas for what might need to be communicated to participants when they stop taking part in a clinical trial or other study**

| **Number** | **Topic** | **Notes/comments** |
| --- | --- | --- |
| **Functional elements** | | |
| 0.1 | Date (i.e. date on letter/message) |  |
| 0.2 | Clear title/subject line on message |  |
| 0.3 | Statement about availability of information in other languages/formats |  |
| 0.4 | "Personal" elements in message e.g. participant name, clinician signature, handwritten signature - or personalisation e.g. details of how many visits done, samples provided etc |  |
| **Participation status** | | |
| 1.1 | Confirmation of which elements of participation have stopped and which have not / confirmation of participant's current status in the trial (including if lost contact) |  |
| 1.2 | Reason why some/all participation stopped if it was not the participant's choice |  |
| 1.3 | Confirmation of date of last involvement |  |
| **Addressing emotions about end of participation** | | |
| 2.1 | Thank you message | i.e. thanking the participant for their contribution to the study |
| 2.2 | Recognition of any negative feelings at stopping early (guilt, disappointment, 'letting down', abandonment, anxiety) |  |
| 2.3 | Message about how stopping early is totally fine and understandable |  |
| 2.4 | Acknowledgement of individuals' contributions (/importance) | Slightly different to ‘thank you’ – emphasising that ‘your contribution has made a difference’ |
| 2.5 | Message encouraging feelings of pride about participation / no need to feel the opposite [/recognition of other positive feelings] |  |
| 2.6 | Suggestion to thank those who helped you get through the trial |  |
| **Rights and choices** | | |
| 3.1 | Reminder of rights around withdrawal / other participant rights | i.e. you have the right to stop taking part in any part of the study at any time, without giving a reason and without your care being affected  Reminder of info given before initial consent |
| 3.2 | Reminder of limitations on what can be 'withdrawn' e.g. samples already used, devices already implanted | It isn’t always possible to ‘undo’ all elements of participation – examples given here are about implanted devices (if that was part of the research) or biological samples that have already been used (they can’t be un-used)  Reminder of info given before initial consent |
| 3.3 | What choices are still available (and which are not e.g. if treatment cannot restart) |  |
| 3.4 | Reminder of importance of participants | Slightly different to the point 2.4 above, this would be a general point about the importance of participants in trials. It is in this section as it could be relevant information to inform participant choices |
| 3.5 | Reminder about why retention is important / impact of missing data | As per discussions in PeRSEVERE, it would likely be worded positively, i.e. the research will get a more reliable result if we are able to collect more of the information we planned to collect (rather than negative, i.e. if we cannot collect more information about you, the research will be harmed)  Reminder of info given before initial consent |
| 3.6 | Possibility of participants making further contributions to the study / benefits of this (to individual) (i.e. with reduced commitment) |  |
| 3.7 | Invitation to clarify wishes regarding change in participation / how to notify about changed wishes in future |  |
| 3.8 | Invitation to give reason for stopping participation and why this is helpful to the research | Reminder of info given before initial consent |
| **Regaining contact if lost** | | |
| 4.1 | If lost contact, message expressing concern for wellbeing |  |
| 4.2 | If lost contact, request to get in touch with researchers/clinical team |  |
| 4.3 | If lost contact, facilitator/mechanism to get in touch e.g. enclosing a stamped addressed envelope |  |
| 4.4 | Ways the researchers may try to regain contact if lost / intention to continue trying to regain contact | Reminder of info given before initial consent |
| **Practical arrangements around stopping participation** | | |
| 5.1 | How to safely stop trial medications/ interventions (e.g. tapering treatment, risks of sudden withdrawal) - including procedures required to ensure safety | Not applicable to all studies |
| 5.2 | Existence of a final study visit / exit interview (e.g. for safety when stopping treatment, or final bit of data collection, or to provide support to patient) | Reminder of info given before initial consent  Not applicable to all studies |
| 5.3 | Information on returning leftover treatment | Not applicable to all studies |
| 5.4 | Picking up belongings if participation involved staying somewhere (may be specific to healthy volunteer trials) | Only applies where participation involves inpatient stay |
| 5.5 | Required/requested paperwork around early stopping (e.g. “withdrawal form” for purpose of confirming or clarifying wishes) | Reminder of info given before initial consent  May not apply to all studies |
| 5.6 | Impact of stopping early on prior agreements/obligations or reminder of these [in hypothetical situation where contracts apply] | Only applies in the hypothetical situation where participant has made formal agreement with researchers rather than just given consent (an idea put forward by some bioethicists) – unlikely to apply to **any** studies but included in this list for completeness  Reminder of info given before initial consent |
| **Resolving deception** | | |
| 6.0 | Information about study withheld at initial consent (i.e. 'resolving deception') - e.g. with expression of regret for deception and opportunity to withdraw consent if applicable | In some studies, it is necessary for the research objectives to withhold some information or even give incorrect information prior to initial consent. This is mostly relevant to e.g. psychology experiments, but it could at least theoretically apply to some clinical trials, e.g. where the fact of patients’ treatment being decided by randomisation (rather than a clinical choice) might not be disclosed to patients. In studies involving this sort of deception or withheld information, there is usually an obligation to ‘debrief’ i.e. provide all the information to the participants at the end of the study. Participants usually have the opportunity to withdraw their data at this point if they are not happy with having participated in the study.  Only relevant to studies where some deception used/information withheld |
| **Arrangements for care and treatment after the trial** | | |
| 6.1 | Reassurance about stopping participation early not affecting care overall | Reminder of info given before initial consent |
| 6.2 | Reminder about any possible negative consequences on health/medical care from stopping early (e.g. no longer having access to study treatment) | Reminder of info given before initial consent |
| 6.3 | Arrangements for post-trial care (including 'transition' to it) | Best provided by clinical staff rather than trial sponsor |
| 6.4 | What information will be passed to other healthcare professionals (e.g. GP) to support post-trial care / who else notified about early stopping |  |
| 6.5 | Information to know before trying other treatments | Best provided by clinical staff rather than trial sponsor |
| 6.6 | Post-trial access to the study treatment (either via the study or through other means; including if study treatment not available anywhere) | Reminder of info given before initial consent |
| 6.7 | Reminder of treatment details (e.g. drug name etc / specific details of dose etc) | Reminder of info given before initial consent |
| 6.8 | Whether or not the study drug may be licensed, when/how/etc |  |
| 6.9 | Reminder of longer-term safety risks or potential side-effects (expected or unexpected): specific follow-up of these; how to manage and report them | Reminder of info given before initial consent |
| 6.10 | Updated safety data about treatment received (e.g. affecting risk-benefit ratio; or possibility of that info coming later/how it would be communicated) |  |
| 6.11 | Support/advice for managing health (e.g. with chronic condition, availability of healthcare services) |  |
| 6.12 | Reasons for any post-stopping processes/ requirements | “Requirements” as in the other points mentioned here – point here just means reasons why the processes are needed  Reminder of info given before initial consent |
| 6.13 | Whether or not study team will make any further contact / reasons why they might |  |
| **Available support** | | |
| 7.1 | Availability of counselling/support specifically about end of study | i.e. about stopping participation early |
| 7.2 | Opportunities to communicate with other participants/patients | i.e. within the same study  Not always possible or appropriate |
| 7.3 | Tips for adjusting to post-trial life/schedule |  |
| **Information about treatment allocation** | | |
| 8.1 | Treatment allocation (including help in understanding this) | i.e. what treatment was received, if this was previously not disclosed to the participant  Only for blinded studies |
| 8.2 | Reassurance of importance of contribution for those on placebo/similar | Only for blinded studies |
| 9.0 | Requests to limit disclosing information about study to others (e.g. treatment allocation, deception) | If the researchers decide it is appropriate to let some participants know what treatment they were getting while some others still don’t know, then it might be worth mentioning this and requesting that participants do not share this information (and explain why it would be helpful if they don’t)  The “please do not share” message may apply to other topics as well, e.g. the one about deception |
| **Incentives/payments** | | |
| 9.1 | Information about incentives/ reimbursement (e.g. which ones apply, how to obtain, any withheld until end of study) | Reminder of info given before initial consent  Only for studies where payments/ incentives are available |
| **How data have been/will be used** | | |
| 10.1 | Reminder of how data have been/will be used (“transparency information”) - including limitations on rights | “Limitations on rights” includes that data collected already for the study will be retained by the researchers – if participants could get it deleted, this would harm the research. This position is backed up by data protection laws.  Reminder of info given before initial consent |
| 10.2 | Reminder of rights/limitation on rights regarding data deletion / what will happen to data already collected | Could be combined with the point above; slightly different in that that is general about how data have been and will be used, and this point is specifically about the data deletion issue  Reminder of info given before initial consent |
| 10.3 | Reminder that confidentiality will be maintained / security | Reminder of info given before initial consent |
| 10.4 | Reminder/ information about further uses of study data, including sharing or regulatory submissions | “Sharing” here means for further research purposes; “regulatory submissions” means to regulatory agencies like the MHRA for the purposes of getting treatments approved for general use.  Reminder of info given before initial consent |
| 10.5 | Plans to continue accessing routine data and existence of opt-out | “Routine data” here means data collected by the NHS as part of patients’ routine visits, rather than specifically for a study. If a study participant stops attending study-specific visits but carries on with routine visits, the data from the routine visits might still be useful for the study.  Reminder of info given before initial consent  Depends on what participant has said they want to stop |
| 10.6 | Reminder about limitations on ability to opt out of some further data collection (e.g. about safety of treatments) | Some information may continue to be collected indefinitely in order to meet legal requirements around monitoring the safety of treatments. Where this applies, it would routinely be explained to patients before they agree to take part in a clinical trial.  Reminder of info given before initial consent |
| **Biological samples** | | |
| 11.1 | Reminder of what biological samples given | Reminder of info given before initial consent  Only for studies involving biological sample collection |
| 11.2 | What will happen to biological samples collected / options in this | Reminder of info given before initial consent  Only for studies involving biological sample collection |
| **Trial status and information about trial’s results** | | |
| 12.1 | Status of trial (including if now closed, recruitment numbers etc) |  |
| 12.2 | How other participants are doing (generally in terms of health, side effects) | Likely only available through talking with clinical team (i.e. trial sponsor unlikely to be able to comment on this, for various reasons) |
| 12.3 | When and how trial results will be available (including how to opt out of receiving) | This means the overall results of the trial (rather than any personal results – see below about that)  Reminder of info given before initial consent |
| 12.4 | Information given on preferences for receiving study results / how to update these | i.e. if participants have been asked already if they want to receive the results, this would be a reminder of what they said, including about how they would like to receive results (by email, post etc) |
| 12.5 | Benefits and risks of receiving overall trial results | “Risks” here might include anything that could be difficult about receiving the trial results – for example if the trial shows that a new treatment does not work, this might be disappointing. This content might well have already been given prior to initial study consent. |
| 12.6 | Opportunity to be involved in interpreting or communicating results (PPI work) | For example, if there are plans to hold a meeting of participants near the end of the study in order to discuss the study results and what they mean. |
| 12.7 | Responses to media coverage about trial or intervention | Might apply in some high-profile studies or studies involving high-profile or controversial treatments. |
| 12.8 | Results from other relevant trials / up-to-date information about this trial's intervention(s) | i.e. information from other trials about the same treatment |
| **Individual health results** | | |
| 13.1 | Individual health results (from the trial or incidental findings), including support for deailng with these | This includes things like how each participant’s health has changed over the course of the study, and also ‘incidental findings’ i.e. health information that the study was not looking for but has found out about, such as genetic results or unexpected results from scans or other tests.  Reminder of info given before initial consent  May not apply to all studies |
| 13.2 | Possibility of disclosing individual results to family members (genetics) | Some genetics ‘results’ (e.g. finding out that a participant is at risk of a certain genetic condition) might impact family members. Studies where this issue may be relevant might have a policy about notifying family members or not (particularly in the situation where a study participant dies while taking part). This topic in this list would be a reminder of this policy.  Reminder of info given before initial consent  May not apply to all studies |
| **Giving feedback** | | |
| 14.1 | Opportunity to give feedback about the trial (including how study could be improved) |  |
| 14.2 | Opportunity to give general feedback on research participation experience | e.g. through surveys like NIHR’s patient research experience survey <https://www.nihr.ac.uk/patients-carers-and-the-public/i-want-to-learn-about-research/participant-in-research-experience-survey.htm> |
| 14.3 | Complaints procedure | May be particularly relevant to participants stopping early if they have stopped due to a negative experience  Reminder of info given before initial consent |
| **Other research opportunities** | | |
| 15.1 | Opportunity to take part in qualitative interviews (separate, related research) | This is similar to the point about feedback above, but slightly different; this would be any interview-based research linked to the main study, for example to explore patients’ experiences |
| 15.2 | Information about future/other trials (including to take part in, or opportunities to be notified of future studies/retention of details for this purpose) |  |
| 15.3 | Suggestion to encourage others to take part (specific to paid research) | May only apply to healthy volunteer research |
| **Opportunities for research involvement** | | |
| 16.1 | PPI opportunities | For example, an invitation to get involved in other studies as a patient contributor or to learn more about PPI in general |
| **Further information** | | |
| 17.1 | Study team contact details (e.g. in case of questions or requests for more information, or to get in touch for lost contact people) |  |
| 17.2 | Request for updated contact details/need to keep them updated | i.e. from the participant |
| 17.3 | FAQs | i.e. Frequency Asked Questions, if there are any |
| 17.4 | Study website link |  |
| 18.1 | Study arm-specific information (if applicable) |  |
| **Reminders about the trial** | | |
| 19.1 | Study name and identifiers | Reminder of info given before initial consent |
| 19.2 | Sponsor details | i.e. which organisation is responsible for the running of the study  Reminder of info given before initial consent |
| 19.3 | Name/details of specific trial site | Could be in the form of e.g. headed paper  Reminder of info given before initial consent |
| 19.4 | Trial registry details | This means the public registry showing details of the study – all studies have to register before they begin and have to publish their results on the registry within set timelines at the end of the study. Giving the link allows participants to be aware of this public information about the study.  Reminder of info given before initial consent |
| 19.5 | Reminder of study purpose, methods and aims (and what it hopes to change/improve) | Reminder of info given before initial consent |
| 19.6 | Reminder that study has appropriate approvals | Reminder of info given before initial consent |
| 19.7 | Reminder of PPI in study | i.e. reminder that patients are involved in designing and overseeing the study |
| 19.8 | Reminder of ethics committee review/oversight of study | Reminder of info given before initial consent |
| 19.9 | Chief/main investigator details | Reminder of info given before initial consent |
| 19.10 | Conflict of interest information | i.e. if the researchers are being paid by certain companies to do the study  Reminder of info given before initial consent |
| 19.11 | Reminder about initial information given / check still remember key points / offer to receive another copy | This would include how to obtain another copy of the pre-study information if it’s needed |
| 19.12 | Links to more information (e.g. about the study, the clinical area or anything else) |  |
