# Supplementary material for: How can we support research participants who stop taking part? Communications guidance developed through public-researcher collaboration
Source: Res Involv Engagem. 2024 Apr 18;10:39. doi: 10.1186/s40900-024-00572-4 (PMC11025252; doi:10.1186/s40900-024-00572-4)
Supplement: Supplementary file 4 — Additional file 4. Short form GRIPP2 reporting checklist. [file 40900_2024_572_MOESM4_ESM.docx]

**Supplement 4: short form GRIPP2 reporting checklist**

| **Section and topic** | **Item** | **Reported on page No** |
| --- | --- | --- |
| 1: Aim | Report the aim of PPI in the study | 8 |
| 2: Methods | Provide a clear description of the methods used for PPI in the study | 11-12 |
| 3: Study results | Outcomes—Report the results of PPI in the study, including both positive and negative outcomes | 13-17 |
| 4: Discussion and conclusions | Outcomes—Comment on the extent to which PPI influenced the study overall. Describe positive and negative effects | 15-17, 19-20 |
| 5: Reflections/critical perspective | Comment critically on the study, reflecting on the things that went well and those that did not, so others can learn from this experience | 19-20 |

From: [GRIPP2 reporting checklists: tools to improve reporting of patient and public involvement in research](https://researchinvolvement.biomedcentral.com/articles/10.1186/s40900-017-0062-2)
